# The link between the risk of cardiovascular diseases and the intake of different types of dietary carbohydrates in Iranian adults

**DOI:** 10.1097/XCE.0000000000000311

**Published:** 2024-10-16

**Authors:** Nazanin Beheshti, Aryan Tavakoli, Zahra Saeedirad, Zahra Mousavi, Narjes Nooriani, Khadijeh Abbasi Mobarakeh, Zahra Mahmoudi, Majid Kamali, Saeideh Mohammadi, Seyed Ali Namakian, Parsa Bahmani, Sara Khoshdooz, Maryam Gholamalizadeh, Saeid Doaei, Akram Kooshki

**Affiliations:** a Blood Transfusion Research Center, High Institue for Research and Education in Transfusion Medicine; b Department of Clinical Nutrition, School of Nutritional Sciences and Dietetics, Tehran University of Medical Sciences; c Department of Clinical Nutrition and Dietetics, Faculty of Nutrition and Food Technology, Shahid Beheshti University of Medical Sciences; d School of Nursing and Midwifery, Shahed university, Tehran; e Department of Community Nutrition School of Nutrition and Food Science, Isfahan University of Medical Sciences, Isfahan; f Deparment of Nutrition science and research Branch, Islamic Azad University, Tehran; g Nutrition and Food Sciences Research Center, School of Nutrition and Food Sciences, Isfahan University of Medical Sciences, Isfahan; h Zanjan University of Medical Sciences, Zanjan; i Faculty of Medical Sciences and Technologies, Azad Islamic University of Medical Sciences,; j Department of Community Nutrition and Dietetics, Faculty of Nutrition and Food Technology, Shahid Beheshti University of Medical Sciences, Tehran; k Faculty of Medicine, Guilan University of Medical Sciences, Rasht; l Cancer Research Center, Shahid Beheshti University of Medical Sciences; m Department of Community Nutrition, National Nutrition and Food Technology Research Institute, Faculty of Nutrition Sciences and Food Technology, Shahid Beheshti University of Medical Sciences, Tehran; n Non-Communicable Diseases Research Center, Sabzevar University of Medical Sciences, Sabzevar, Iran

**Keywords:** Cardiovascular diseases, dietary carbohydrates, starch, sucrose

## Abstract

**Background:**

The risk of cardiovascular diseases (CVDs) may be influenced by dietary carbohydrates. The aim of this study was to investigate the link between CVDs and the intake of carbohydrates.

**Methods:**

In this cross-sectional study, data was extracted from the Prospective Epidemiologic Research Studies in Iran (PERSIAN) cohort in Sabzevar, Iran. A total of 4241 adults, including 1535 patients with CVDs and 2706 people without CVDs, were included. A validated 237-item food frequency questionnaire was used to estimate the intake of different types of dietary carbohydrates.

**Results:**

A positive association was found between stroke and dietary intake of starch (OR = 1.108; 95% CI, 1.005–1.220; *P* = 0.039). Additionally, a negative association was found between stroke and dietary intake of sucrose (OR = 0.97; 95%CI, 0.94–0.99; *P* = 0.037). No association was found between other types of CVDs and the intake of different types of carbohydrates.

**Conclusion:**

This study provided some evidence for the association between CVDs and different types of dietary carbohydrates. Consumption of starch may increase the risk of stroke, while a higher intake of sucrose may decrease the risk of stroke. Further studies are warranted.

## Introduction

Cardiovascular disease (CVD) refers to a group of medical conditions that influence the heart and blood vessels, including coronary artery disease, heart failure, stroke, and peripheral artery disease [1]. Research indicates that over half a billion people worldwide are affected by CVDs [2]. In 2021, CVDs accounted for 20.5 million deaths, constituting almost one-third of the global mortality [2]. Moreover, the prevalence of CVDs has been consistently rising over the last several decades, with a notable 60% increase in morbidity rates recorded between 1990 and 2021 [2]. In Iran, CVD has grown to be the leading cause of mortality, accounting for 46% of all deaths and contributing 20–23% to the overall disease burden [3].

Previous research identified several key risk factors associated with the development and progression of CVDs including elevated blood pressure, elevated levels of low-density lipoprotein cholesterol (LDL-C) [4], diabetes, smoking and secondhand smoke exposure, obesity, physical inactivity, and unhealthy dietary habits [5]. The risk factors may be varied among different types of CVD. For example, coronary artery disease (CAD) is one of the major CVDs, and the risk factors of CAD include diabetes, hypertension, smoking, hyperlipidemia, obesity, homocystinuria, and psychosocial stress [6]. Also, elevated plasma levels of LDL-C and hepatic secretion of very low-density lipoprotein (VLDL) increase the risk of atherosclerotic CVD [7]. Diet components affect the level of many of these risk factors both directly and indirectly [8].

Carbohydrates are one type of macronutrients in the diet that play a crucial role in providing energy for the body and are an essential component of a healthy diet [9]. Carbohydrates are the main component of the energy intake of Iranians dietary pattern, and its consumption reaches about 60% of the calories consumed and even more in some Iranian populations [10]. Carbohydrates can be classified into three distinct types, included monosaccharides, oligosaccharides, and polysaccharides [11]. Complex carbohydrates (polysaccharide) are reported to be associated with a reduced risk of CVD, whereas sugars (monosaccharide and disaccharide) are positively associated with an increased risk [12,13]. From another point of view, some studies have examined the impact of carbohydrates on CVD by considering their glycemic index (GI). Several studies have shown that consuming carbohydrates with a low GI leads to reduced postprandial glucose and insulin levels, enhanced insulin sensitivity, and improved lipid profiles [14,15]. In addition, diets with a high GI may promote lipogenesis and lead to larger adipocytes, while diets with a low GI have been found to prevent these reactions. Therefore, the GI as a carbohydrate-related index in our diet appears to have a significant impact on our metabolism, potentially influencing the risk of CVDs, diabetes, and obesity [14,15].

On the other hand, a study using UK Biobank data investigated the impact of various types of dietary carbohydrates on CVD risk and concluded that there was no clear association between total carbohydrate intake and CVD outcomes [16]. In other views, a recent meta-analysis indicates a heightened risk of CVD with high-carbohydrate intake is influenced by the geographic variations, for example, an increased risk was observed in Asian populations, while no associations were seen in the Americas and Europe [17]. There are few studies on the association of different types of CVDs with different types of carbohydrates. So, the present study seeks to fill this gap by evaluating each type of carbohydrate individually and elucidating their impact on CVDs to gain a deeper understanding of their role in CVD progression.

## Methods

In this cross-sectional study, we extracted the required data from the Prospective Epidemiologic Research Studies in Iran (PERSIAN) cohort in Sabzevar City, Iran. The Persian cohort study protocol and other information regarding the objectives, design of the study, sample size, sampling methods, participant selection, quality assurance, quality control, and outcomes of interest can be found elsewhere [18]. The present study included a total of 4241 adults aged 35 years, comprising 1535 patients with CVDs [529 individuals with heart diseases, including myocardial ischemia and myocardial infarction (MI), and 1006 individuals with hypertension (HTN)] along with 2706 individuals without CVDs. Sampling was carried out using the census method. The inclusion criteria involved individuals of Iranian nationality residing within the study area, aged 35–70 years, providing consent to participate in the study, and had been diagnosed with heart diseases, ischemia, MI, and HTN for the case group. The exclusion criteria included individuals who were unwilling to continue the participation, those who had communication difficulties that hindered their ability to answer study questions, individuals with mental illnesses, intellectual disabilities, hearing or visual impairments, and any other disorder that could potentially interfere with the research process, incomplete medical records, the presence of certain severe underlying conditions such as diabetes, diagnosis of heart diseases occurring more than 2 months before study enrollment, and the utilization of slimming supplements, steroids, cholesterol-lowering drugs, barbiturates, and carbamazepine.

### Data collection

With the agreement of the relevant authorities at the PERSIAN Cohort Center in Sabzevar, data were originally gathered using valid and reliable questionnaires developed to acquire information from participants in PERSIAN cohort sites. Participants were interviewed at the cohort site by a trained professional following an invitation. The PERSIAN cohort general questionnaire was applied to gather comprehensive information on demographic characteristics (such as gender, age at enrollment, household income, marital status, and education), lifestyle factors (including dietary intake, level of physical activity, smoking, and drinking habits), and medical history (such as the use of specific medications such as statins, presence of CVDs and diabetes). The level of physical activity and the metabolic equivalent of task (MET) was measured using a validated physical activity questionnaire [19]. One MET equals approximately 3.5 ml of O_2_ per kg of body weight which is the amount of energy required to sleep.

### Blood pressure assessment

HTN was assessed by the measurement of the participants’ blood pressure by trained personnel. Three sets of SBP and DBP readings were taken using a validated sphygmomanometer (Omron M10-IT model, Omron Healthcare, Kyoto, Japan). The average of the last two measurements was used for each participant. The mean arterial pressure (MAP) was calculated as DBP + [0.333 * (SBP–DBP)], where DBP represents diastolic blood pressure and SBP denotes systolic blood pressure. The measurements were conducted on the participant’s dominant arm while sitting, after a minimum of 5 min of rest. A cuff of suitable size was used by measuring the circumference of the upper arm according to the guidelines of the European Society of Hypertension [20].

### Anthropometric measurements

The weight and height of the participants were assessed while wearing lightweight clothing and without shoes. The weight was measured in kilograms with a precision of 0.1 kg using a mechanical column scale (Seca 755, Germany), while the height was measured in centimeters with a precision of 0.1 cm using a stadiometer (Seca 204, Germany). The participants’ BMI was computed by dividing their weight (in kg) by the square of their height (in m) [21].

### Dietary assessment

The nutritional data for the Persian cohort was sourced from a validated and reliable Food Frequency Questionnaire (FFQ), which included 237 food items common in the Iranian diet. This comprehensive FFQ was used to document participants’ regular consumption over the previous year [22]. The intake of various carbohydrates such as glucose, fructose, galactose, sucrose, maltose, and lactose were analyzed using Nutritionist IV software. For this analysis, the carbohydrate content of each food item was first calculated, and then total intake of each carbohydrate was estimated based on the quantity consumed by each individual.

### Statistical analysis

The data analysis was conducted using SPSS software version 21 (SPSS Inc., Chicago, USA). The participants’ sociodemographic, anthropometric, and medical indicators were described using descriptive statistics, including SD and mean for quantitative data, and number and percentage for qualitative variables. Independent *T*-test and chi-square test were used to compare the quantitative and qualitative variables, respectively. Multiple logistic regression models were used to adjust the confounding variables, including age, gender, education, physical activity, BMI, smoking, using alcohol, underlying diseases, and the intake of calorie, protein, fat, total carbohydrate, and other types of carbohydrates. The link between CVDs as categorical outcomes and dietary carbohydrates as quantitative continuous exposures was assessed using the odds ratio (OR) with a 95% confidence interval (CI). To avoid implausible assumptions regarding risk variation within categories [23], the exposure was considered as a quantitative continuous variable. A significance threshold of *P* < 0.05 was used for all statistical computations.

## Results

Characteristics of participants are presented in Table 1. In this study, the patient with CVD was older (54.19 ± 8.03 vs. 47.20 ± 8.23 years; *P* < 0.001), and had a higher BMI (29.38 ± 4.77 vs. 27.70 ± 4.62 kg/m^2^; *P* < 0.001), right SBP (124.59 ± 19.13 vs. 110.36 ± 14.16 mmHg; *P* < 0.001), right DBP (77.40 ± 11.39 vs. 69.73 ± 9.29 mmHg; *P* < 0.001), triglyceride (TG) (157.04 ± 92.84 vs. 142.09 ± 105.71 mg/dL; *P* < 0.001), and diabetic history (26.7% vs. 8.9%; *P* < 0.001) compared with the controls group. The CVD group had lower physical activity (37.55 ± 7.17 vs. 39.08 ± 7.99 kcal/kg/d; *P* < 0.001) and LDL-C (106.41 ± 35.69 vs. 111.92 ± 32.72 mg/dL; *P* < 0.001) than the control group.

**Table 1:** The general characteristics of the participants

	CVD group (*n* = 1535)	Healthy group (*n* = 2706)	*P* value^a^
Age (y)	54.19 ± 8.03	47.20 ± 8.23	<0.001
Females (*n*, %)	909 (59.2%)	1458 (53.9%)	<0.002
Males (*n*, %)	626 (40.8%)	1247 (46.1%)	<0.002
Last education			<0.01
Up to diploma	999 (65.1%)	1632 (60.3%)	
Graduated	328 (21.4%)	413 (15.3%)	
Postgraduated	208 (13.5%)	661 (24.4%)	
Physical activity (MET/d)	37.55 ± 7.17	39.08 ± 7.99	<0.001
Height (cm)	160.84 ± 9.14	162.76 ± 9.19	<0.001
Weight (kg)	75.98 ± 13.52	73.34 ± 13.35	<0.001
BMI (kg/m^2^)	29.38 ± 4.77	27.70 ± 4.62	<0.001
Right SBP (mmHg)	124.59 ± 19.13	110.36 ± 14.16	<0.001
Right DBP (mmHg)	77.40 ± 11.39	69.73 ± 9.29	<0.001
TG (mg/dl)	157.04 ± 92.84	142.09 ± 105.71	<0.001
CHOL (mg/dl)	189.65 ± 43.26	192.54 ± 38.88	0.044
HDL-C (mg/dl)	52.11 ± 10.84	52.50 ± 10.56	0.283
LDL-C (mg/dl)	106.41 ± 35.69	111.92 ± 32.72	<0.001
Drink alcohol (yes) (*n*, %)	100 (6.5%)	195 (7.2%)	0.463
Has diabetes (yes) (*n*, %)	409 (26.7%)	241 (8.9%)	<0.001
Has job (yes) (*n*, %)	468 (30.5%)	1274 (47.1%)	<0.001
Smoking (yes) (*n*, %)	563 (36.7%)	1396 (51.6%)	<0.001

CVD, cardiovascular diseases; CHOL, cholesterol; HDL-C, high-density lipoprotein cholesterol; LDL, low-density lipoprotein; MET, metabolic equivalent of task; TG, triglyceride.

a *P* values were obtained using independent *T*-test and chi-square test.

Table 2 shows the intake of calorie-adjusted macronutrients and carbohydrates among the case and control groups. The case group exhibited a higher intake of carbohydrates (331.14 ± 32.36 vs. 328.85 ± 29.95 g/d; *P* = 0.034), starch (10.63 ± 10.11 vs. 9.93 ± 9.18 g/d; *P* = 0.008), and glucose (23.34 ± 11.15 vs. 21.86 ± 10.72) and lower intake of energy (2392.61 ± 779.02 vs. 2541.04 ± 14.9 kcal/d; *P* < 0.001) and sucrose (35.48 ± 22.14 vs. 37.35 ± 21.75 g/d; *P* = 0.012) compared to the control group. However, no significant difference was found between the intake of protein, fat, fructose, galactose, lactose, and maltose between the groups.

**Table 2:** Calorie intake and calorie-adjusted intake of macronutrients among the participants

	CVD group (*n* = 1535)	Healthy group (*n* = 2706)	*P* value^a^
Protein (g/d)	62.79 ± 8.52	63.33 ± 8.18	0.059
Total fat (g/d)	51.88 ± 14.46	52.12 ± 13.01	0.610
Carbohydrate (g/d)	331.14 ± 32.36	328.85 ± 29.95	0.034
Energy (kcal/d)	2392.61 ± 779.02	2541.04 ± 14.9	<0.001
Starch (g/d)	9.93 ± 9.18	10.63 ± 10.11	0.008
Sucrose (g/d)	35.48 ± 22.14	37.35 ± 21.75	0.012
Glucose (g/d)	23.34 ± 11.15	21.86 ± 10.72	0.001
Galactose (g/d)	0.16 ± 0.14	0.15 ± 0.16	0.101
Fructose (g/d)	27.03 ± 12.54	25.49 ± 12.39	0.904
Lactose (g/d)	3.69 ± 4.40	3.43 ± 3.65	0.073
Maltose (g/d)	0.41 ± 0.44	0.41 ± 0.48	0.583

CVD, cardiovascular diseases.

a *P* values were obtained using independent *T*-test.

**Fig. 1:**
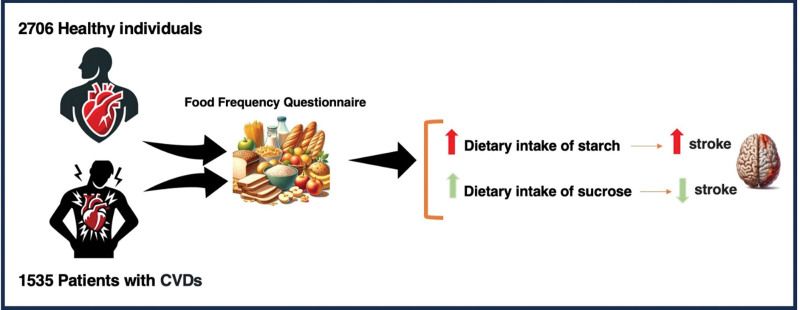
The association between cardiovascular diseases (CVDs) and the intake of different types of dietary carbohydrates.

Regarding the association between different types of CVDs with different types of carbohydrates, a marginal positive association was observed between stroke and the dietary intake of starch (OR = 1.108; 95% CI, 1.005–1.220; *P* = 0.039) (Table 3). Additionally, a marginal negative association was identified between stroke and dietary sucrose intake (OR = 0.97; 95%CI, 0.944–0.998; *P* = 0.037). No significant association was observed between stroke and the dietary intake of glucose, galactose, dextrose, fructose, and lactose. Moreover, there was no observed association between other types of CVDs and the intake of various types of carbohydrates (Fig. 1).

**Table 3:** The association of cardiovascular diseases and different types of dietary carbohydrates

	HTN		CI		MI		Stroke		All CVDs	
	OR (95% CI)	*P* value	OR (95% CI)	*P* value	OR (95% CI)	*P* value	OR (95% CI)	*P* value	OR (95% CI)	*P* value
Total carbohydrate	1.002 (0.998–1.005)	0.56	1.001 (0.998–1.004)	0.56	1.001 (0.993–1.009)	0.81	1.008 (0.999–1.017)	0.09	1.002 (0.998–1.005)	0.091
Starch	1.008 (0.968–1.049)	0.707	0.993 (0.949–1.040)	0.780	0.984 (0.908–1.066)	0.696	1.108 (1.005–1.220)	0.039	1.013 (0.977–1.051)	0.469
Sucrose	0.999 (0.992–1.005)	0.685	0.999 (0.993–1.006)	0.808	0.999 (0.987–1.011)	0.842	0.971 (0.944–0.998 )	0.037	0.999 (0.993–1.004)	0.607
Glucose and dextrose	1.015 (0.953–1.080)	0.642	1.023 (0.955–1.095)	0.517	1.007 (0.892–1.138)	0.907	1.009 (0.805–1.264)	0.936	1.028 (0.972–1.087)	0.333
Galactose	1.021 (0.545–1.910)	0.32	0.551 (0.092–3.313)	0.515	1.059 (0.928–2.445)	0.32	1.405 (0.933–2.115)	0.11	1.002 (0.998–1.005)	0.291
Fructose	0.989 (0.935–1.045)	0.692	0.981 (0.923–1.043)	0.542	0.983 (0.881–1.096)	0.754	0.994 (0.818–1.209)	0.955	0.983 (0.936–1.034)	0.509
Lactose	0.985 (0.944–1.028)	0.484	1.036 (0.996–1.079)	0.082	1.048 (0.988–1.111)	0.119	0.825 (0.65–1.035)	0.096	0.988 (0.952–1.025)	0.526

Adjusted for age, gender, education, physical activity, BMI, smoking, using alcohol, underlying diseases, and the intake of calorie, protein, fat, and total carbohydrate, and other types of carbohydrates.

CI, cardiac ischemia; CVD, cardiovascular disease; HTN, hypertension; MI, myocardial infarction.

## Discussion

The study’s findings demonstrated a borderline positive association between stroke and the consumption of starch in diet. Furthermore, a marginal negative association was identified between stroke and dietary intake of sucrose. Several previous studies have shown the influence of high-carbohydrate diets, particularly those abundant in refined carbohydrates, on a heightened prevalence of CVDs [24]. Diets rich in refined carbohydrates have been associated with developing atherogenic dyslipidemia, which is characterized by high levels of TG, low levels of HDL cholesterol, and higher amounts of LDL particles [25,26].

Khan *et al*. [27] in a systematic review and meta-analysis found that overall sugar intake and fructose were associated with an increased risk of death from CVD, but there was no such association with CVD incidence or with the intake of added sugars. In line with this study, sucrose intake was found to be beneficial, reducing the risk of CVD mortality. Dose-response analysis suggested that there is a harmful level of consumption of total sugars and fructose in CVD death, while sucrose consumption showed a beneficial effect across all levels of intake [27]. Another research demonstrated a positive relationship between the consumption of carbohydrates derived from refined starches and added sugars and the risk of coronary heart disease (CHD) [12]. Also, another study, from the National Health and Nutrition Examination Survey, 2003–2014, revealed that people with starchy snacks after main meals had greater risk of CVD and all‐cause mortalities [28]. Some other studies reported an adverse association of sugar-sweetened beverages with incidents of heart disease and stroke [29–32]. Jo *et al*. [17] investigated that a high-carbohydrate diet may elevate the risk of CVD, particularly in Asian populations, probably because of genetics and a higher intake of carbohydrates. Another investigation showed high intake of carbohydrates, especially refined carbohydrates with a high GI and glycemic load (GL), is associated with a higher risk of stroke [33]. In contrast with our study, the Japanese cohort study suggested that in Japanese men, high starch intake may reduce CVD-related mortality risk, while high sugar intake including glucose, fructose, and sucrose may increase mortality risk. The effects in women were less pronounced, with only free sugars showing a significant impact [34]. Other studies revealed that a high GI did not significantly increase stroke risk. However, a high GL was found to be a risk factor for stroke. There was no substantial association found between high-carbohydrate intake and stroke risk [35]. The contradiction in the obtained results may be caused by the effect of gene variations on the relationship between heart diseases and dietary carbohydrate intake [36]. For example, high intake of carbohydrates in people with polymorphism in the FTO gene may affect the expression of some genes involved in metabolism [37].

A diet high in certain types of starch, particularly those that are processed quickly by the body into glucose, can lead to rapid increases in blood sugar levels [38]. Also, studies indicated that an overabundance of carbohydrates may impair the liver’s capacity to metabolize carbohydrates into fat and impede the production of advantageous high-density lipoprotein cholesterol (HDL-C) [39–42]. This disruption can lead to increased levels of TG and reduced levels of HDL-C, both of which are notable variables that increase the risk of metabolic diseases such as insulin resistance and metabolic syndrome [43]. These conditions are major predictors of a high 10-year risk for CVD [44]. However, in countries that consume high-carbohydrate diets, there might be lower consumption of other essential macronutrients, such as protein and fats, which leads to nutritional imbalance and this imbalance may elevate the risk of developing CVD [45–48]. Conversely, sucrose, which is composed of glucose and fructose, may not have the same immediate impact on blood sugar levels when consumed in moderation. Unlike starch, sucrose’s fructose component is metabolized differently and does not cause a rapid increase in blood glucose. Consequently, moderate sucrose consumption might not lead to the same level of blood sugar spikes, potentially reducing the risk of developing insulin resistance and subsequent cardiovascular issues [38].

The strengths of this research were conducting a comprehensive study considering different types of CVD and different types of dietary carbohydrates on a relatively high sample size. However, some limitations of this study need to be mentioned. One limitation of this study was the use of a self-reported questionnaire for collecting dietary data with a high probability of underreporting of obese people and overreporting of underweight people. In addition, this was a cross-sectional study which limited our ability to determine causality between carbohydrate consumption and CVD risk factors. Another weakness of the current study is the lack of information on all types of monosaccharides, disaccharides, polysaccharides and dietary fibers, so we could not organize different carbohydrates based on mono, di, and polysaccharide. Further studies are needed to increase our knowledge of the interaction between different types of carbohydrates and CVD.

### Conclusions

In summary, this study provided some evidence for an association between CVDs and different types of dietary carbohydrates. Consumption of starch may increase the risk of stroke. Also, a negative association was found between stroke and dietary intake of sucrose. Future longitudinal studies on the different types of dietary carbohydrates and the incidence of CVDs are needed. If the results of the present study are confirmed, considering that the major part of daily caloric intake comes from dietary carbohydrates, it can play an important role in providing appropriate dietary recommendations to people at risk of heart disease.

## Acknowledgements

We thank all the participants in this study for their good cooperation. This paper was taken from the approved research project of Shahid Beheshti University of Medical Science.

Funding for this study was provided by Sabzevar University of Medical Sciences, Sabzevar, Iran (402205).

The ethics committee of Sabzevar University of Medical Sciences, Sabzevar, Iran approved the study (Ethics Code: IR.MEDSAB.REC.1400.021). All study participants signed a written informed consent.

Institutional consent forms were used in this study.

The datasets utilized and/or analyzed in the present study are accessible upon reasonable request from the corresponding author.

N.B., M.T., Z.S., Z.M., N.N., and S.D. designed the study, and were involved in the data collection, analysis, and drafting of the manuscript. K.H.A.M., Z.M.A., M.K., P.B., S.K.H., S.D., and M.G.H. were involved in the design of the study, analysis of the data, and critically reviewed the manuscript. All authors read and approved the final manuscript.

### Conflicts of interest

There are no conflicts of interest.
